# Design of an immunogen containing multidimensionally conserved and immunogenic parts of the HIV proteome

**DOI:** 10.1371/journal.pcbi.1014632

**Published:** 2026-08-14

**Authors:** Eric Wang, Arup K. Chakraborty

**Affiliations:** 1 Institute for Medical Engineering and Science, Massachusetts Institute of Technology, Cambridge, Massachusetts, United States of America; 2 Department of Chemical Engineering, Massachusetts Institute of Technology, Cambridge, Massachusetts, United States of America; 3 Department of Physics, Massachusetts Institute of Technology, Cambridge, Massachusetts, United States of America; 4 Department of Chemistry, Massachusetts Institute of Technology, Cambridge, Massachusetts, United States of America; 5 Ragon Institute of Massachusetts General Brigham, Massachusetts Institute of Technology, and Harvard University, Cambridge, Massachusetts, United States of America; University of Rochester Medical Center, UNITED STATES OF AMERICA

## Abstract

Despite decades of research, an effective HIV vaccine or cure has not yet been developed. An important reason is the virus’s mutability, which allows it to rapidly evade human immune responses. Often, the virus also takes advantage of epistatic pathways to evolve escape mutations through compensatory effects that allow maintaining its fitness. We used a fitness landscape of HIV, which explicitly considers interactions between mutations, to design an immunogen that contains parts of the proteome that are “multidimensionally” conserved. Based on inferred fitness landscapes, these regions are predicted to be under mutational constraints. Our immunogen also incorporates predicted immunogenic regions and offers broad coverage for the Caucasian population. We further investigate the relative contributions of various proteins in our design and compare properties with other immunogens. We hope that our proposed immunogen will serve as a candidate for experimental evaluation in pre-clinical studies in T cell-based vaccine or immunotherapy contexts.

## Author summary

Developing an effective HIV therapeutic or prophylactic vaccine has been a major challenge due to the virus’s ability to rapidly mutate and escape human immune responses. Furthermore, even when the immune system forces the virus to mutate at sites that result in a fitness penalty, secondary mutations elsewhere, known as compensatory mutations, can evolve to partially restore its replicative capacity. To counter this, we designed a vaccine immunogen for eliciting T cell responses using a computational model that identified parts of virus proteins that are both conserved and where compensatory mutations are difficult to evolve. If T cells target these regions, the virus may be effectively trapped, as mutating to escape the T cell response would hurt its replicative capacity. We also aimed to select regions that can be recognized by T cells in a large portion of the population. We compare our design to previous candidate immunogens. We hope this computationally designed vaccine will serve as a candidate for future pre-clinical testing in T cell-based HIV vaccines and immunotherapies.

## 1. Introduction

The emergence of human immunodeficiency virus (HIV) has had devastating impacts on global health with over 40 million deaths [1]. While the development of antiretroviral therapies has led to a significant reduction in HIV-related mortality, the ongoing unavailability of these therapies in certain regions remains a concern [2]. Furthermore, antiretroviral therapies do not eliminate the virus and are not curative, meaning that the virus will rebound if infected individuals are taken off therapy [3]. Long-term side effects of these drugs can also be challenging for some [4]. An alternative is the development of an effective vaccine or immunotherapy to cure or prevent disease, which despite progress remains elusive [5]. Achieving such a cure will likely require a multi-pronged approach that leverages both cellular and humoral immunity.

The primary challenge lies in the virus’s remarkable mutability, with mutation rates ranging from 10^-5^ to 10^-3^ per base pair per replication, depending on the method used [6,7]. Such high mutability allows the virus to continually evade human immune responses, even within an individual. However, there is optimism that vaccines designed to elicit immune responses targeting conserved epitopes can still be effective against mutable viruses. For instance, a key objective of antibody-based vaccines is to induce broadly neutralizing antibodies that focus on the conserved CD4 binding site, critical for the virus’s entry into host cells [8–10].

Another vaccination/therapeutic approach aims to trigger an effective T-cell response that targets viral peptides (epitopes) presented on human leukocyte antigen (HLA) proteins on the surfaces of infected cells. When exposed to these viral epitopes, CD8 T-cells can kill infected cells, which could potentially control infection or remove infected cells over time [11]. A sustained, effective T-cell response is hypothesized to potentially lead to a functional cure [12]. However, the T-cell response is also subject to immune escape [13], suggesting that vaccines should focus T-cell responses on epitopes where escape mutations are unlikely because they reduce viral fitness.

One approach is to target epitopes that exhibit conservation at the level of single residues, as such conservation may signify functional importance, akin to the CD4 binding site. Ongoing investigations aim to design immunogens incorporating conserved epitopes such as tHIVconsvX [14] and others [15–17]. However, it is also known that HIV takes advantage of epistasis, or interactions between multiple mutations, to make compensatory mutations that allow the virus to simultaneously evade the immune response and maintain its fitness [18]. One approach to address this challenge that has been pursued is to use a sequence-based model of HIV fitness that accounts for epistasis, and to then identify epitopes that are both conserved and unlikely to contain compensatory interactions with other parts of the sequence [19–25]. This type of conservation is referred to as multidimensional conservation. In another approach, Gaiha et al. applied network analysis (that was previously used to study proteins that include influenza proteins [26,27]) to HIV protein structures and identified highly networked epitopes that would be difficult to mutate [28].

**Fig 1:**
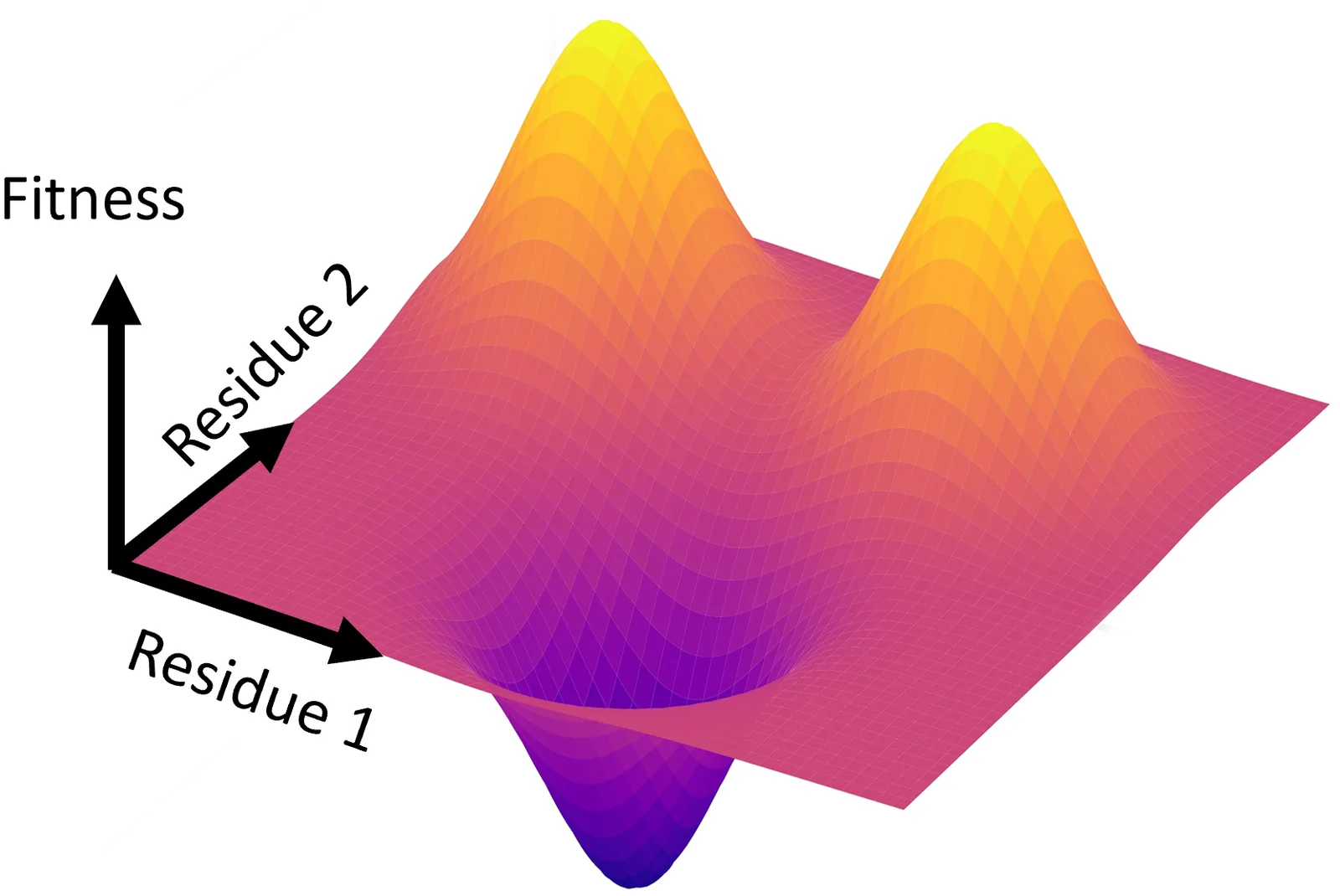
Illustration of the fitness landscape of a viral protein composed of two residues, where each axis represents possible amino acids for each residue and the vertical dimension represents viral fitness.

Several efforts have led to inferring the HIV fitness landscape from thousands of HIV sequences [20–23,29], using a model rooted in statistical physics that explicitly accounts for both single mutations and interactions between pairs of mutations (Fig 1). Since the data used to train the model comes from existing HIV sequences, the inferred model is the prevalence landscape of HIV, meaning that the model outputs the probability of observing a given sequence in the human population. Using the prevalence landscape as a fitness landscape assumes that virus strains that appear more frequently in circulation are more fit. In a single person, certain viral strains may be more prevalent because of their ability to evade human immune pressure rather than their intrinsic fitness for infection and replication.

However, evidence has been provided suggesting that this effect is not significant at the level of the population of circulating viruses [21,23,25,30]. This is because of several reasons, including the fact that HIV has not been subject to an effective population-wide immune response (“elite controllers” are a very small fraction of the population [31]). Comparisons of predictions from the inferred prevalence landscape with in vitro replication measurements and in vivo escape time measurements show strong correlation [19,21–23], suggesting that the prevalence and intrinsic fitness landscapes are statistically similar. In these comparisons, experimental in vitro replicative capacity assays were employed to measure the relative growth kinetics quantified in cell culture for diverse HIV mutants generated via site-directed mutagenesis. These data showed a strong agreement with predictions based on the sequence energies computed by the model. Similarly, the model’s predictions have been validated against longitudinal clinical data by mapping the initial HIV-specific CD8 + T cell responses in acutely infected individuals. By tracking how the virus evolved to evade a specific initial T cell response over time, escape mutations predicted by the model to have higher fitness costs were found to take significantly longer to emerge in vivo. Furthermore, the escape times themselves strongly correlated with those predicted using the fitness landscape and an evolutionary model. Although extended escape times do not guarantee that these epitopes are intrinsically protective, the time required to evolve escape mutations is a highly relevant metric for therapeutic vaccines. Thus, our past work suggests that T-cell responses directed at multidimensionally constrained epitopes result in delayed viral escape.

Additionally, there is orthogonal structural evidence supporting the fitness landscape. When the fitness landscape for gp160 was inferred, the results were compared to structural data [22]. The strongest inferred couplings were highly predictive of the residue contacts observed in the crystal structure of the SOSIP trimer and other crystal structures. The residues that were inferred to be strongly coupled, but not in contact, were primarily found in the V2 or V4 loops or the CD4 binding site. The strongly coupled non-contact residues in the V2 loop and CD4 binding site could reflect documented conformational changes in the V2 loops when the viral spike interacts with CD4, and interactions with CD4 and other co-receptors. This analysis indicated that the inferred strongly coupled residues identified protein contacts and other features within the functional gp160 trimeric spike, rather than just contacts within isolated monomers. That is, the inferred fitness landscape captures features of the underlying physical and structural architecture that constrains the evolution of the virus.

Theoretical studies suggested reasons for why the prevalence landscape inferred from sequence data was predictive of intrinsic fitness in the case of HIV [25]. The main reasons are: 1] Because of the great diversity of HLA alleles in the human population, no region of HIV’s proteins is targeted by T cell responses of a significant fraction of humans. 2] With the exception of the small number of elite controllers, T cell responses mounted by humans are not effective. Thus, unlike influenza, the population of circulating HIV strains has not evolved in highly directed ways due to effective population-level human immunity.

In previous work, Murakowski et al. used the fitness landscape to identify multidimensionally conserved regions of the HIV proteome and created an adenovirus-vectored vaccine containing this immunogen [24]. They then showed that this vaccine was as immunogenic as vaccines containing whole HIV protein antigens in rhesus macaques. This study was promising but also had several limitations. Most notably, the adenovirus vector imposed constraints on the length of the immunogen which necessitated the inclusion of some epitopes that were not multidimensionally conserved. The adenovirus vector also could not use non-immunogenic linkers, which required concatenating blocks together and introducing junctional epitopes. Besides these limitations related to the adenovirus vector, they also did not consider epitope immunogenicity in their design.

**Fig 2:**
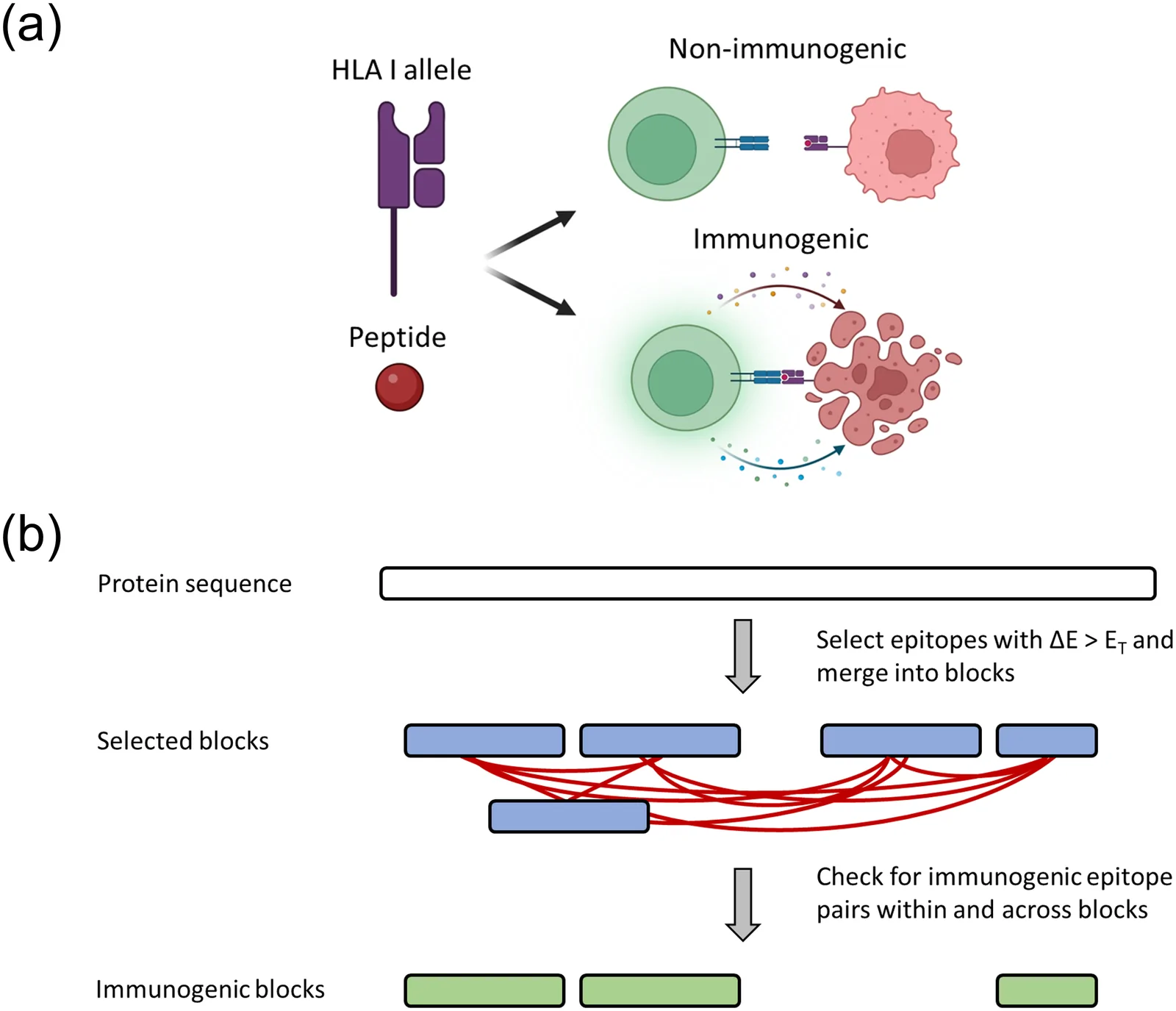
(a) Illustration of the immunogenicity predictor, which inputs an HLA I allele and a peptide sequence and outputs whether the peptide is immunogenic to the HLA allele (Created in BioRender. Wang, E. (2026) https://BioRender.com/elncib5). (b) Illustration of the immunogen design algorithm, starting with fitness cost-based selection and followed by immunogenicity based selection. In fitness cost-based selection, ΔE is the pairwise fitness cost (Equation 7) and E_T_ is the fitness cost threshold above which viral escape is unlikely, as suggested by comparison of model predictions and clinical data.

In recent years, mRNA vaccines have emerged as a new technology capable of providing strong protection against a variety of pathogenic viruses [32,33]. Given the efficacy and relative lack of constraints with mRNA vaccines, we have designed a new immunogen to address the limitations noted above. Using a previously developed immunogenicity predictor by Gao et al. (Fig 2), we also selected regions of the proteome that are immunogenic for HLA haplotypes found in the Caucasian population. We show that our immunogen selects for regions of the HIV proteome that are not only conserved in single residues but also reduced in compensatory interactions and enhanced in antagonistic interactions. Also, our immunogen is predicted to be immunogenic and provides broad coverage to the Caucasian population. We then compare our immunogen to previous immunogens for T cell-based vaccines. Throughout this work, we propose our computationally designed immunogen as a candidate for a therapeutic vaccine, specifically designed to elicit T-cell responses that kill infected cells, which can be used in combination with complementary antibody-based approaches.

## 2. Results

### 2.1 Immunogen design algorithm

As depicted in Fig 2, our immunogen design algorithm proceeds in two stages. Initially, we select blocks from the consensus sequences of various HIV proteins based on their multidimensional conservation, which we quantify as defined in Section 2.1.1. From these selected blocks, we use our immunogenicity predictor to calculate which blocks add immunogenic epitope pairs when included. These further selected blocks are thus both multidimensionally conserved and immunogenic, and they form the final immunogen.

#### 2.1.1 Fitness cost based selection.

To define multidimensionally conserved regions of the HIV proteome, we use the inferred fitness landscape of HIV [19–23], which is a function that provides the fitness of a viral strain relative to the consensus sequence given its protein sequence. The functional form of the fitness landscape is as follows:


$$\begin{array}{c} \begin{array}{c} \text{f}(\text{s})=\frac{\text{exp}\left(-\text{E}(\text{s})\right)}{\text{Z }} \end{array} \end{array}$$ (1)

$$\begin{array}{c} \text{E}(\text{s})=-\sum_{\text{i}=\text{1}}^{\text{N}}{\text{h}}_{\text{i}}^{\alpha }{\text{s}}_{\text{i}}^{\alpha }-\sum_{\text{i}<\text{j}}^{\text{N}}{\text{J}}_{\text{ij}}^{\alpha \beta }{\text{s}}_{\text{i}}^{\alpha }{\text{s}}_{\text{j}}^{\beta } \end{array}$$ (2)

where s is the sequence of the HIV protein, and f is the fitness or probability of observing s. Following statistical mechanical terminology, E is the “energy” and Z is the “partition function” which normalizes f. ${\text{h}}_{\text{i}}^{\alpha }$ (also called a field) is an inferred parameter that quantifies the fitness cost of mutating a residue i from the consensus amino acid to the α amino acid, and ${\text{J}}_{\text{ij}}^{\alpha \beta }$ (called a coupling) is an inferred parameter that quantifies the fitness cost of evolving two mutations at residues i and j to amino acids α and β, respectively. Negative values of ${\text{h}}_{\text{i}}^{\alpha }$ correspond to large fitness costs, suggesting that residue i is more conserved. Negative values of ${\text{J}}_{\text{ij}}^{\alpha \beta }$ suggest that mutations α and β at residues i and j have an antagonistic interaction and a higher fitness cost than summing the effects of each individual mutation, while positive values of ${\text{J}}_{\text{ij}}^{\alpha \beta }$ suggest a compensatory interaction and lower fitness cost. The values of ${\text{h}}_{\text{i}}^{\alpha }$ and ${\text{J}}_{\text{ij}}^{\alpha \beta }$ were inferred from HIV sequences in previous work [20–23,29]. The HIV proteins for which the fitness landscape was inferred are indicated in S1 Table. All proteins except for p15 from Pol have been inferred in previous work.

With the fitness landscape, we first calculate a quantity referred to as the pairwise fitness cost for every pair of nonoverlapping epitopes in the HIV proteome. Epitope pairs in our analysis are defined as any pair of non-overlapping 9-mer epitopes and are not restricted to occurring within the same block. Pairs can therefore occur within a block or across different blocks of the same protein within the immunogen. Short blocks will typically not contain multiple non-overlapping epitopes, and any pairs would form with other blocks.

The pairwise fitness cost can be thought of as the average change in fitness due to making two mutations in the pair of epitopes. The cost also accounts for interactions with various sequence backgrounds (the rest of the sequence that is not the two epitopes). The equations describing this calculation are described in Methods, and we briefly describe the calculation here. For a given pair of mutations, Equation 1 is used to calculate the change in fitness due to these mutations compared to the unmutated sequence, and this change in fitness is then averaged over an ensemble of sequence backgrounds weighted by the probabilities with which they are in circulation (as predicted by the fitness landscape). This is then further averaged over all possible pairs of mutations that could arise in the epitope pair, thus leading to the pairwise fitness cost.

Our use of averaging over different sequence backgrounds is informed by previous work that illustrated the importance of the sequence background on viral escape [19]. As an example, one person had HIV strains with compensatory mutations in the sequence background with respect to the targeted epitope, and this person exhibited viral escape in 122 days whereas another person with fewer compensatory and more antagonistic sequence background mutations never exhibited viral escape within the observation period of 1103 days. Our calculation of the pairwise fitness cost accounts for variations in the sequence background as described, and so epitope pairs with many potential compensatory mutations in background sites would correspond to a low pairwise fitness cost.

With the pairwise fitness costs of every pair of epitopes in the HIV proteome, we proceed with the fitness cost selection algorithm. First, we begin the immunogen by selecting the epitope pair with the largest pairwise fitness cost. We then grow our immunogen by iteratively adding new epitopes. At each iteration, we select the next epitope to add by choosing the one with the highest average pairwise fitness cost with the epitopes already in the immunogen. Before adding the new epitope to the immunogen, we check if any of its pairwise fitness costs fall below a threshold value of ${\text{E}}_{\text{T}}=\text{8}.\text{5}$. If none fall below ${\text{E}}_{\text{T}}$, then we add the new epitope to the immunogen and continue the process, but if one does fall below ${\text{E}}_{\text{T}}$, then we do not add the new epitope and stop the algorithm.

The value of ${\text{E}}_{\text{T}}$ was informed by clinical data of viral escape in humans [19]. It was observed that it was difficult to escape from immune responses targeting epitopes with fitness costs above 8.5 predicted by the inferred fitness landscape. Another issue is that Pol is less immunogenic than other HIV proteins. Since it is less immunogenic, there is less pressure to evolve escape mutations and thus less observed sequence variation in Pol proteins. To account for this, we use a stricter threshold of 12.75 for Pol proteins as in Murakowski et al.

After the fitness cost selection part of the algorithm is complete, we merge our list of epitopes into blocks to consider for our final immunogen. Note that two 9-mer epitopes can be merged into a larger 10-mer block if they overlap by 8 residues without adding or removing any 9-mer epitopes. This can be done successively, merging many overlapping epitopes to form larger blocks, which is how we form blocks out of our list of epitopes (Fig 2).

This process builds on work from Murakowski et al. but differs in a few ways: (1) Most importantly, Murakowski et al. introduced several subsequent steps after selecting blocks with high pairwise fitness costs to meet the constraints of the adenovirus vector, but this required lowering the fitness cost threshold and introducing junctional epitopes. We have kept only the first step to maximize the pairwise fitness costs of our immunogen, and we do not introduce junctional epitopes; (2) Murakowski et al. compared the average pairwise fitness cost to the threshold value instead of the minimum pairwise fitness cost to obtain larger blocks for the adenovirus vector. This included some epitope pairs with very low pairwise fitness cost, so we have adjusted to comparing the minimum pairwise fitness cost; (3) Murakowski et al. considered epitopes to be 11-mers, but we consider epitopes to be 9-mers in this work because 9-mers are more relevant for immunogenicity, which we later consider in Section 2.1.2; (4) Murakowski et al. did not include gp120 because the fitness landscape of gp120 was not yet available, but we have included it in our immunogen.

#### 2.1.2 Immunogenicity based selection.

The blocks selected from fitness cost selection are further selected for immunogenicity. Here, we make use of the immunogenicity predictor from Gao et al. [34], which classifies a given peptide sequence and HLA I allele as immunogenic or not immunogenic. The predictor was inspired by predictors of neoantigen immunogenicity for cancer immunotherapy [35] and takes the following form:


$$\begin{array}{c} \begin{array}{c} \text{A}(\text{s},\text{ M})=\frac{\text{1}}{\text{binding}(\text{s},\text{ M})}*\text{R}(\text{s},\text{ pathogen})*\text{R}(\text{s},\text{ self}) \end{array} \end{array}$$ (3)

$$\begin{array}{c} \text{R}(\text{s},\text{pathogen})=\sum_{\text{e}\in \text{pathogens}}\theta \left(|\text{e},\text{s}|-{\text{a}}_{\text{pathogen}}\right) \end{array}$$ (4)

$$\begin{array}{c} \text{R}(\text{s},\text{self})=\sum_{\text{e}\in \text{selfs}}\theta \left(|\text{e},\text{s}|-{\text{a}}_{\text{self}}\right) \end{array}$$ (5)

where A(s,M) is the immunogenicity metric, s is the sequence of the peptide, M is the HLA allele, $\frac{\text{1}}{\text{binding}(\text{s},\text{M})}$ is a measure of the probability that the peptide s can be presented on HLA allele M and is calculated using netMHCpan [36], R(s,pathogen) is the number of known pathogenic peptides with alignment scores greater than a threshold value of ${\text{a}}_{\text{pathogen}}$ to s, and R(s,self) is the number of known self-peptides with alignment scores greater than a threshold value of ${\text{a}}_{\text{self}}$ to s. In Equations 4 and 5, |e,s| is the BLOSUM62-based Smith-Waterman alignment score between s and e (either a known pathogenic or self-peptide), and θ is the step function. ${\text{a}}_{\text{pathogen}}$ and ${\text{a}}_{\text{self}}$ are the only parameters of the model and are trained on a dataset of immunogenic and non-immunogenic HIV peptides from people with known HLAs. To determine if a peptide s is immunogenic to HLA M, we calculate the immunogenicity metric A(s,M) and consider the peptide to be immunogenic if A(s,M) is greater than a threshold value of ${\text{A}}_{\text{T}}=\text{7024}$, which was determined by balancing the true positive rate and true negative rate of immunogenicity classification [34]. Note that the immunogenicity predictor explicitly includes terms not only for HLA binding but also sequence similarity to known pathogenic and self peptides, thereby including T-cell recognition as well as HLA binding.

The immunogenicity predictor was evaluated on immunogenicity classification for both HIV epitopes and SARS-CoV-2 epitopes to show out-of-distribution generalization. The predictor yielded an AUROC around 0.71 for HIV epitopes and predicted CTL responses to some SARS-CoV-2 epitopes that were experimentally validated. To further test the immunogenicity predictor beyond what was reported in Gao et al., we evaluated its predictions on interferon gamma release assays for HIV epitopes from the IEDB database [37] in S1 Fig. This evaluation yields an AUROC of 0.72, near the reported value of 0.71, further validating the capabilities of the predictor. That said, collection of additional immunogenicity data and further improvements in immunogenicity predictors would be useful for many applications including immunogen design.

To identify the prevalence of HLAs in the population, we obtained HLA I haplotypes and their frequencies in the Caucasian population from the Allele Frequency Net Database [38]. We selected the Caucasian population because the fitness landscape was inferred using HIV clade B sequences, which are more prevalent in European and North American regions [39,40]. It is also possible to use the fitness landscape of clade C viruses, which are more prevalent in southern Africa [40], together with the haplotype distribution of the African population to design a different immunogen. A current limitation is that the fitness landscape for clade C gp120 sequences is not yet available, but this is a promising avenue for future work.

With the haplotype distribution of the Caucasian population and the immunogenicity predictor, we then proceed with immunogenicity based selection. For each protein, we consider the blocks that were obtained from fitness cost based selection (blue blocks in Fig 2). We break all blocks into their 9-mer epitopes and calculate whether each epitope is immunogenic to each HLA allele in the Caucasian haplotype distribution. Then, we consider an epitope to be immunogenic to the haplotype if it is immunogenic to any of the three alleles in that haplotype. With this rule, we determine whether each epitope is immunogenic to each haplotype in the Caucasian haplotype distribution. The use of three alleles per haplotype corresponds to each of the three possible class 1 HLA loci (HLA-A, HLA-B, and HLA-C).

At this stage, we know the immunogenicity of each epitope, but we are more interested in the immunogenicity of epitope pairs because we wish to target immune responses against epitopes with antagonistic fitness interactions. Note that every epitope pair in the blocks from fitness cost based selection has high pairwise fitness costs because of the filtering process in Section 2.1.1, and at this step we wish to maximize the number of pairs that are immunogenic. To do this, we form every pair of nonoverlapping epitopes and consider the pair to be immunogenic if both epitopes are individually immunogenic. We start our immunogen with every block obtained from fitness cost selection, and we remove blocks that do not contribute any immunogenic pairs to any haplotype in the Caucasian haplotype distribution. The blocks that remain constitute our final immunogen (green blocks in Fig 2). The frequencies of each haplotype in the Caucasian population are also used to calculate the average number of targeted epitopes and epitope pairs in the population (Section 2.4).

### 2.2 Immunogen sequence

The sequences of the blocks in our immunogen are shown in Table 1 together with the proteins they come from. We have 25 blocks in total with 8 coming from Gag, 5 from Pol, 9 from Env, and 3 from Vif. In S2 Table, we report the amino acid and nucleotide sequences of immunogens formed by stringing together these fragments with non-immunogenic GGSGGGGSGG linkers [41,42]. The amino acid and nucleotide sequences are taken from the corresponding regions of the HXB2 HIV strain, which we chose to use because the HXB2 strain is known to effectively express viral proteins and is nearly identical to the consensus sequence for the selected blocks. Out of 372 residues, HXB2 only differs from consensus in 2 residues, and the amino acids are biochemically similar (T → S and V → I) (Table 1). We formed a single strand with blocks arranged in their 5’ to 3’ order, and our immunogen sequence does not contain any other regions such as signal processing domains or untranslated regions.

**Table 1:** Amino acid sequences of the blocks in our immunogen and the proteins from which they are derived. In blocks 19 and 22, the residue that differs from the HXB2 amino acid is in bold.

Block ID	Protein	Sequence
1	gag	SPRTLNAWVKV
2	gag	EEKAFSPEVIPMF
3	gag	ALSEGATPQDLNTMLNTVGGHQAAMQMLKETINEEAAEWDR
4	gag	NPPIPVGEIYKRWII
5	gag	GLNKIVRMYSP
6	gag	QGPKEPFRDYVDRFYKTLRAEQA
7	gag	VKNWMTETLLVQN
8	gag	NPDCKTILKALGP
9	pol	TVLDVGDAYFSVPL
10	pol	TPDKKHQKEPPFLWMGYELHPDKWT
11	pol	WTVNDIQKLVGKLNWASQIY
12	pol	YWQATWIPEWE
13	pol	NTPPLVKLWYQ
14	vif	MENRWQVMIVWQVDRMRI
15	vif	TYWGLHTGER
16	vif	WHLGQGVSIEWR
17	env	WVTVYYGVPVW
18	env	ISLWDQSLKPCV
19	env	SLKPCVKLTPLCV**T**L
20	env	FNCGGEFFYC
21	env	DNWRSELYKYKVV
22	env	QAR**V**LAVERYL
23	env	VLSIVNRVRQGYSPLS
24	env	LFSYHRLRDLLLI
25	env	VELLGRRGWE

The positions of our blocks on the HXB2 sequence are also visualized in S2–S5 Figs along with the immunogen of Murakowski et al. [24], tHIVconsvX [14], and highly networked epitopes from Gaiha et al. [28]. Our immunogen, as well as others, contains many regions which are known human epitopes from the CTL A-list [43] and known protective epitopes from Mothe et al. [44].

### 2.3 Immunogen contains conserved parts of the HIV proteome, reduces compensatory interactions, and enhances antagonistic interactions

**Fig 3:**
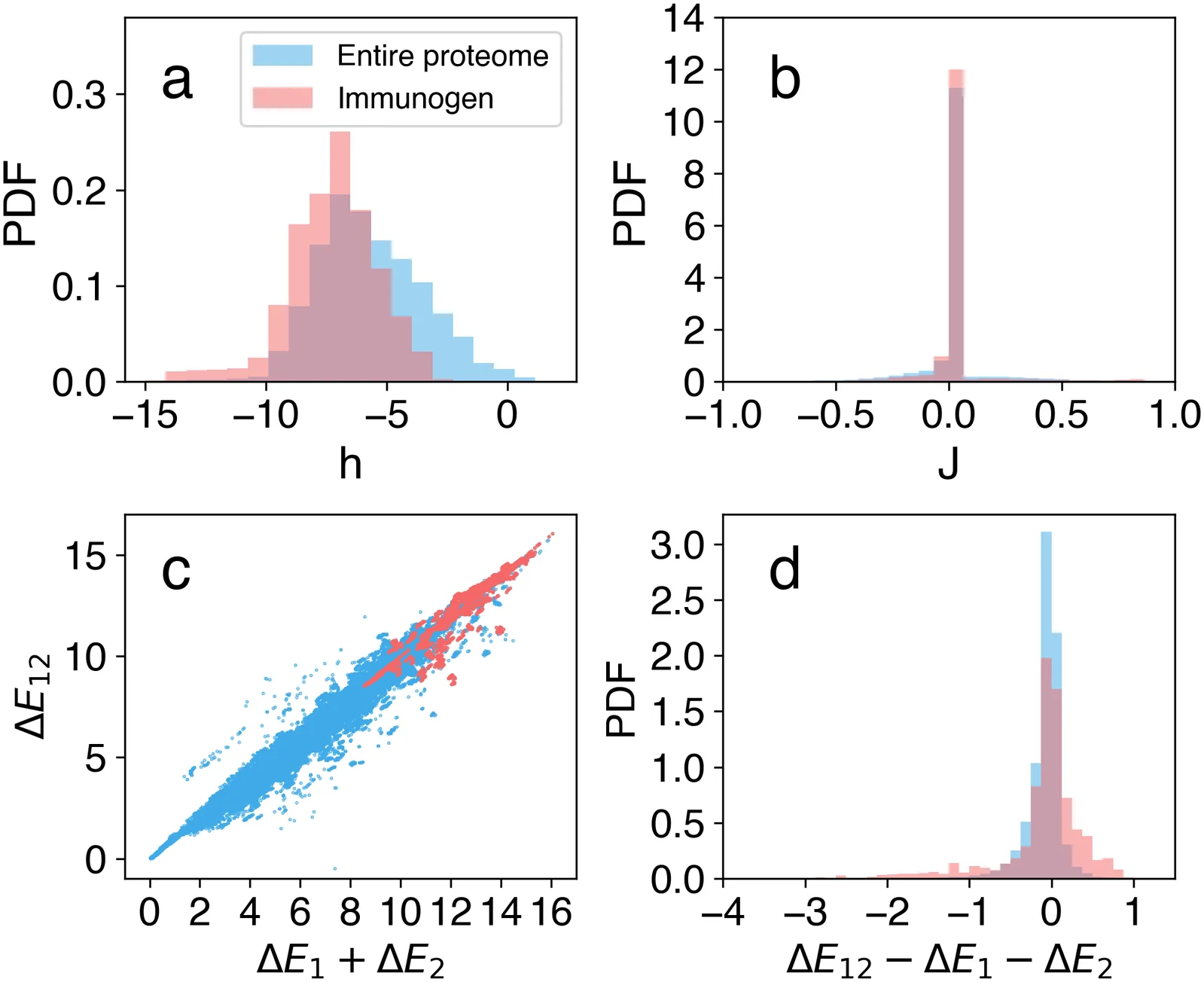
(a) The distribution of fields h in either the entire HIV proteome or in the blocks of our immunogen. (b) The distribution of couplings J in either the entire HIV proteome or in the blocks of our immunogen. (c) Plots of the pairwise fitness cost $\left(\Delta E_{\text{1}\text{2}}\right)$ and sum of individual epitope fitness costs $\left(\Delta E_{\text{1}}+\Delta E_{\text{2}}\right)$ for every epitope pair in either the entire HIV proteome or in the blocks of our immunogen. (d) The distribution of $\Delta E_{\text{1}\text{2}}-\Delta E_{\text{1}}-\Delta E_{\text{2}}$ over every epitope pair in either the entire HIV proteome or in our immunogen. Positive values of $\Delta E_{\text{1}\text{2}}-\Delta E_{\text{1}}-\Delta E_{\text{2}}$ correspond to antagonistic inter-epitope interactions while negative values correspond to compensatory interactions.

Our immunogen contains conserved regions of the HIV proteome, which is expected because conservation at single residues is one component of the fitness cost based selection process. This is visualized in Fig 3a, where we plot the distribution of fields h for both the entire proteome and the blocks of our immunogen. The distribution of our immunogen is shifted towards more negative values, and as previously mentioned, negative values of the fields correspond to more conserved residues. An intuitive way to visualize this is in S2–S5 Figs, where we graph the fraction of sequences from the LANL database (see S1 Text) that are the consensus amino acid for every residue in the proteome and highlight the positions of various immunogens (ours, Murakowski et al., networked epitopes from Gaiha et al., and tHIVconsvX). The blocks in our immunogen consistently align with residues displaying high consensus fractions (the fraction of sequences with the consensus amino acid at that residue), which agrees with the field distribution. Gag is a particularly notable example, as one can see that the breaks between our blocks are at residues with lower consensus fractions, suggesting that our design algorithm detected these sites of reduced conservation and avoided them. This is not the case for the other immunogens. For the Murakowski immunogen, this is attributed to the aforementioned differences in the design algorithm which allowed lower fitness cost regions to accommodate the adenovirus vector constraints. For the epitopes from Gaiha et al., this is likely because the epitopes are identified from structural data rather than sequence data, and they noted that there were sometimes discordances between sequence conservation and network scores. For the tHIVconsvX immunogen, this is likely because longer blocks were used to include more epitopes, with a tradeoff for including some less conserved residues.

In some people, it is possible that their viral strains could have mutations that differ from the immunogen sequence, which would reduce the efficacy of the vaccine. This leads to the question of introducing mutations into the immunogen to account for this. However, given that our blocks correspond to extremely conserved regions where mutations are unlikely, we elected to simply use the consensus sequence. In practice, for therapeutic vaccination it would also be possible to sequence the viral strains prevalent within a person to confirm that they did not already evolve mutations in these epitopes and would be a good candidate for therapy.

It is also noteworthy that tHIVconsvX contains large regions of Pol, whereas the other immunogens, including ours, contain much less. This is likely because Pol is less immunogenic and thus experiences less pressure to mutate, leading to high sequence conservation overall. tHIVconsvX’s focus on conserved residues thus may select for large regions of Pol. However, the Murakowski immunogen and our immunogen imposed a stricter fitness cost threshold for Pol, leading these immunogens to select less Pol than tHIVconsvX. Gaiha et al. identified less of Pol because they used a structure-based approach and were not subject to the potential bias in the conservation of Pol.

On top of single residue conservation, our immunogen also considers interactions between residues using couplings. In Fig 3b, we plot the distribution of couplings for the entire proteome and the blocks of our immunogen. The magnitudes of the couplings are much smaller than the magnitudes of the fields, and it is difficult to make any distinction between the entire proteome and our immunogen based on the couplings since they are both strongly peaked near 0.

However, rather than examining individual couplings, we can assess their collective impact by comparing the pairwise fitness cost (referred to here as $\Delta {\text{E}}_{\text{12}}$) to the fitness costs of individual epitopes ($\Delta {\text{E}}_{\text{1}}$ and $\Delta {\text{E}}_{\text{2}}$). The fitness costs of individual epitopes will encode interactions between the epitope and the sequence background but not inter-epitope interactions, whereas the pairwise fitness cost encodes both (see S2 Text). Nonetheless, targeting individual epitopes is useful since epistatic interactions between the epitope and the sequence background are accounted for. In Fig 3c, we graph $\Delta {\text{E}}_{\text{12}}$ and $\Delta {\text{E}}_{\text{1}}+\Delta {\text{E}}_{\text{2}}$ for every epitope pair in either the entire proteome or the blocks in our immunogen. If inter-epitope interactions were negligible, all data points would align along the diagonal. However, some points clearly deviate from this diagonal, indicating significant inter-epitope interactions. Given HIV’s rapid replication and mutation rates, it is crucial to consider these epistatic pathways, even if they are rare. Also, the blocks of our immunogen have high pairwise fitness costs compared to the overall proteome, which is a direct consequence of the threshold ${\text{E}}_{\text{T}}$ used in the fitness cost selection algorithm.

In Fig 3d, we graph the distribution of $\Delta {\text{E}}_{\text{12}}-\Delta {\text{E}}_{\text{1}}-\Delta {\text{E}}_{\text{2}}$ for the proteome and our immunogen. Positive values of $\Delta {\text{E}}_{\text{12}}-\Delta {\text{E}}_{\text{1}}-\Delta {\text{E}}_{\text{2}}$ indicate antagonistic inter-epitope interactions while negative values indicate compensatory interactions. Across the entire proteome, there is a bias for compensatory inter-epitope interactions, as 66% of the epitope pairs have negative values of $\Delta {\text{E}}_{\text{12}}-\Delta {\text{E}}_{\text{1}}-\Delta {\text{E}}_{\text{2}}$. From an evolutionary point of view, this bias would be useful for mediating viral escape. Within our immunogen, there is an increase in the frequency of positive values and decrease in the frequency of negative values, indicating that antagonistic interactions are enhanced and compensatory interactions are reduced. Overall, our fitness cost selection algorithm effectively selected regions of the proteome that were (1) highly conserved; (2) enhanced in antagonistic interactions and reduced in compensatory interactions to prevent escape through epistatic pathways.

**Fig 4:**
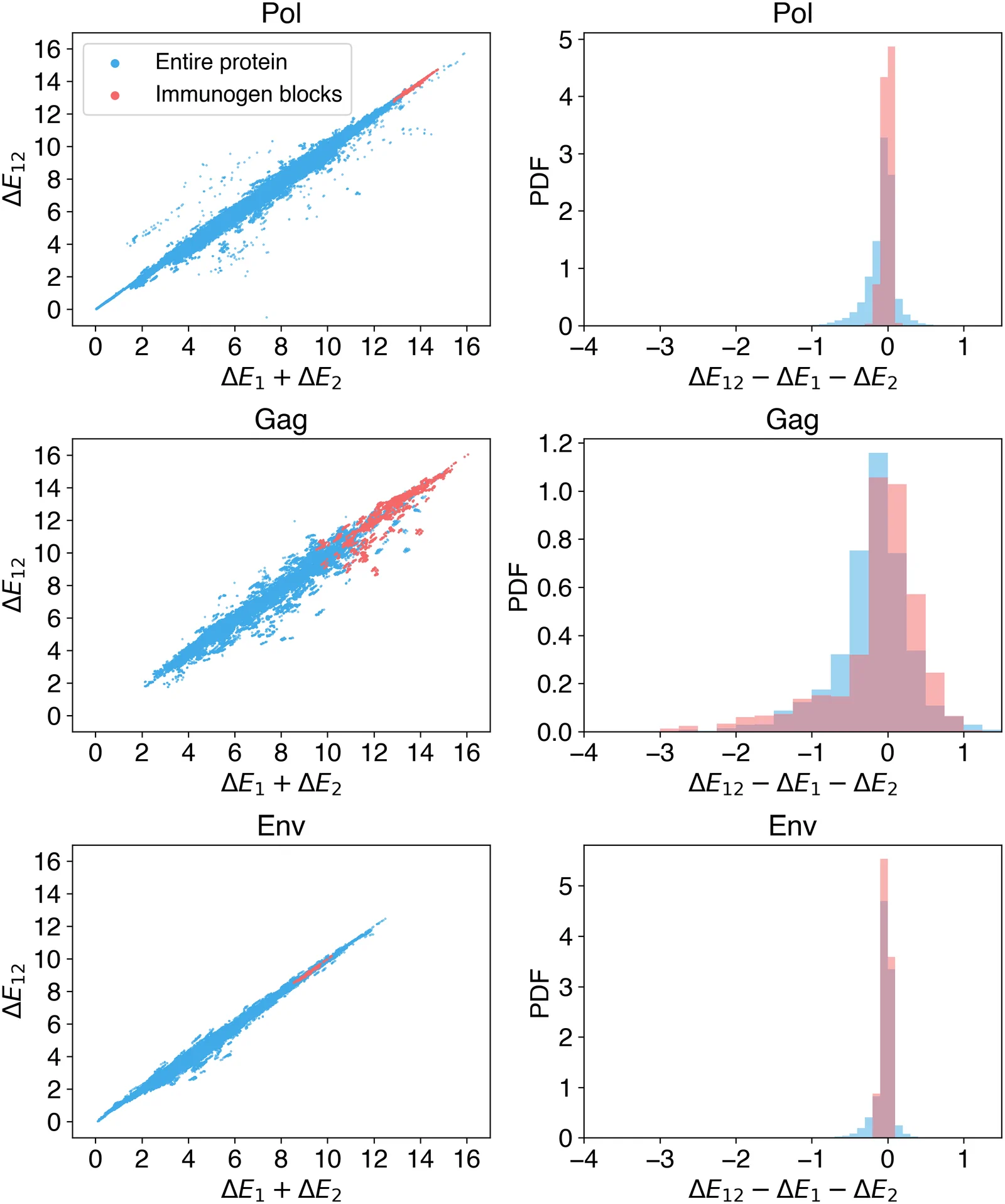
(left) Plots of the pairwise fitness cost $\left(\Delta E_{12}\right)$ and sum of individual epitope fitness costs $\left(\Delta E_{1}+\Delta E_{2}\right)$ for every epitope pair in either entire HIV proteins (Pol, Gag, and Env) or in the blocks of our immunogen from that protein. (right) The distribution of $\Delta E_{\text{1}\text{2}}-\Delta E_{\text{1}}-\Delta E_{\text{2}}$ over every epitope pair in either entire HIV proteins (Pol, Gag, and Env) or in the blocks of our immunogen from that protein. Positive values of $\Delta E_{\text{1}\text{2}}-\Delta E_{\text{1}}-\Delta E_{\text{2}}$ correspond to antagonistic inter-epitope interactions while negative values correspond to compensatory interactions.

We can also examine the properties of the immunogen specific to individual proteins. In S6 Fig, we graph the distributions of fields and couplings for Pol, Gag, and Env. The field distributions reveal that our immunogen selectively includes conserved regions (indicated by negative fields) from each protein, and that Gag has the most negative fields while Env has the least negative. Similar to Fig 3b, the coupling distributions are difficult to interpret, so we look at the values of$\;\Delta E_{\text{1}\text{2}}$ and $\Delta E_{\text{1}}+\Delta E_{\text{2}}$ for each protein in Fig 4. For Env, the values of $\Delta E_{\text{1}\text{2}}$ and $\Delta E_{\text{1}}+\Delta E_{\text{2}}$ are very similar, agreeing with previous observations that the coupling matrix for Env is very sparse [22], whereas there is more deviation in Pol and Gag (Fig 4). This difference is less discernable in the distributions of couplings in S6 Fig, since those distributions are only for individual couplings rather than their collective effects. Looking at the distributions of $\Delta E_{\text{1}\text{2}}-\Delta E_{\text{1}}-\Delta E_{\text{2}}$, it becomes apparent that Gag is responsible for most of the enhancement in antagonistic interactions and also reduces compensatory interactions. In contrast, Pol and Env reduce both compensatory and antagonistic interactions, but they reduce compensatory interactions slightly more.

The fitness costs of previous immunogens (Murakowski et al., tHIVconsvX, and the epitopes from Gaiha et al.) are illustrated in S7 Fig. The pairwise fitness costs for all three tend to be lower than the fitness costs in our immunogen. For tHIVconsvX, this is because it used the conservation of single residues in its design, thereby omitting interactions between epitopes. There is still a noticeable bias towards higher fitness costs in tHIVconsvX since conservation of single residues is one component of the fitness cost. Gaiha et al. used a structural approach based on network theory, so they relied less heavily on sequence conservation, and they demonstrated that many conserved epitopes are poorly networked by their network score. Thus, many conserved epitope pairs with high fitness costs are not included in their epitopes. The Murakowski immunogen is notable because it has similar design algorithm and yet includes low fitness costs. The principal reason for this is that they compared the average pairwise fitness cost instead of the minimum pairwise fitness cost to $E_{T}$, which allowed many pairs with low pairwise fitness costs into the immunogen as long as the average was sufficiently high. Their use of the average was necessary for the adenovirus vector and has been replaced with the minimum in our design, improving the fitness costs of our immunogen. For similar reasons, the distributions of $\Delta E_{\text{1}\text{2}}-\Delta E_{\text{1}}-\Delta E_{\text{2}}$ do not show biases for compensatory or antagonistic interactions in these immunogens.

### 2.4 Immunogen provides broad coverage to the Caucasian population

**Fig 5:**
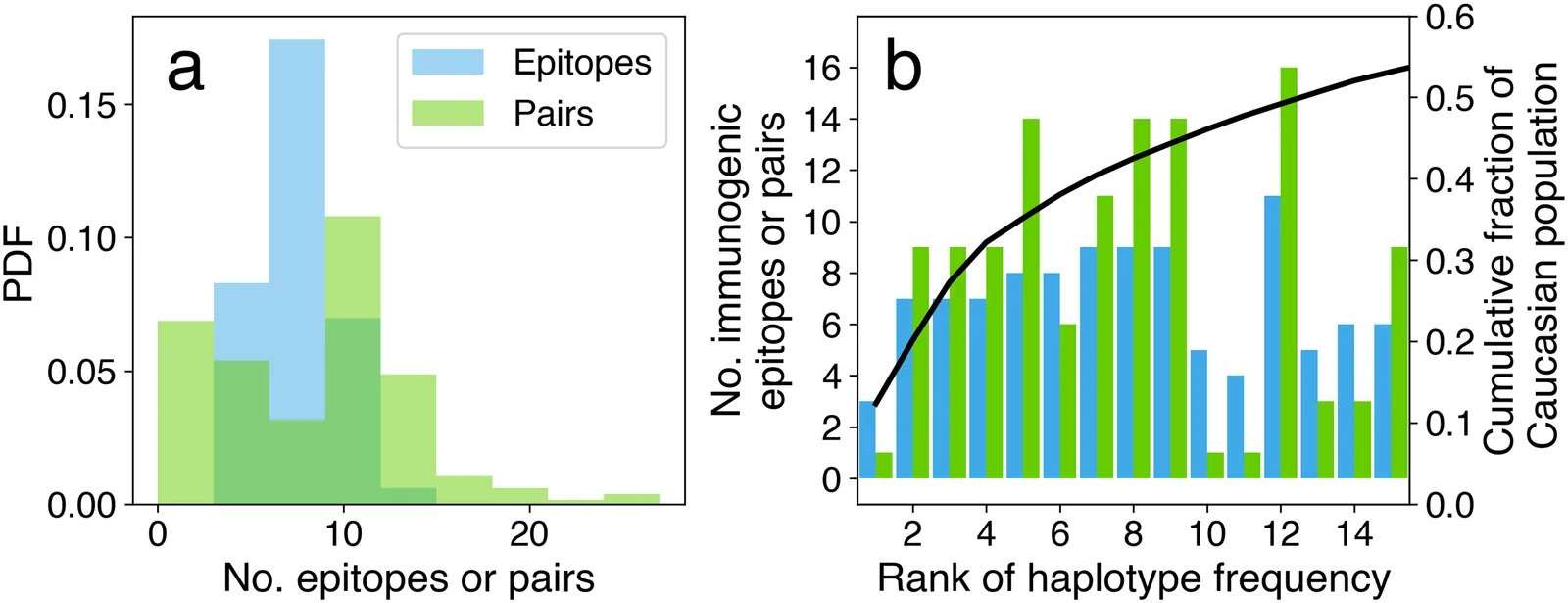
(a) Distributions of the number of immunogenic epitopes and epitope pairs from our immunogen across the Caucasian population. (b) The number of immunogenic epitopes or epitope pairs for the 15 most common haplotypes in the Caucasian population. The black line is the cumulative fraction of the Caucasian population, demonstrating that the 15 most common haplotypes constitute about 50% of the sampled population.

We then assessed the coverage of our immunogen within the Caucasian population. To do this, we utilized the Caucasian haplotype distribution data from the Allele Frequency Net Database [38] and calculated the immunogenicity of each epitope and epitope pair in our immunogen for each haplotype. So, a pair of epitopes can be targeted by an individual with a particular haplotype, and the antagonistic cost of evolving mutations in pairs of epitopes would inhibit viral evolution. Fig 5a graphs the distribution of the number of immunogenic epitopes or epitope pairs across the Caucasian population, demonstrating that a Caucasian individual will have an average of 7 immunogenic epitopes and 8 immunogenic epitope pairs. It has been previously shown that an individual with primary infection of HIV will target an average of 7 epitopes and chronically infected individuals target an average of 20 epitopes [34,45], which suggests that our estimate of 7 epitopes is reasonable. It is worth emphasizing that every haplotype within the sampled Caucasian population targets at least one epitope pair as well as several individual epitopes. Targeting an epitope pair within a protein is important for optimizing inter-epitope interactions, although targeting several individual epitopes across different proteins is very useful too because interactions between the epitopes and the sequence background were also accounted for. Fig 5b plots the number of immunogenic epitopes and epitope pairs for the 15 most common haplotypes, which constitute 50% of the sampled population. Every haplotype has at least 1 immunogenic epitope pair, and some also have up to 16 immunogenic pairs. Given the variability in how many immunogenic pairs will be targeted, identifying strong candidates for therapeutic vaccination through HLA typing may be a prudent strategy for testing in humans.

We break down the contributions from Pol, Gag, and Env in S8 Fig. Gag and Pol contribute the most immunogenic epitopes and pairs, and haplotypes that target many pairs often are a combination of Pol and Gag pairs. For example, the 12^th^ most common haplotype targets 4 Pol and 12 Gag pairs for a total of 16 pairs observed in Fig 5. In contrast, Env contributes relatively few pairs. However, Env was still included in our immunogen because it still contributes important pairs for specific haplotypes. For instance, the 13^th^ most common haplotype targets 3 pairs, one of which comes from Env, so losing this pair by removing Env blocks would significantly impact the immunogen for this haplotype. Similarly, the distribution of individual immunogenic epitopes across haplotypes shows the same pattern (S8 Fig). Gag consistently contributes the largest number of epitopes for most haplotypes, followed by Pol, while Env contributes the fewest. Looking at the 12^th^ most common haplotype, our immunogen contains 6 immunogenic Gag epitopes, which is greater than the 3 Pol epitopes and 1 Env epitope. This is consistent with Env being highly mutable.

Our predictions are also consistent with empirical data: the weighted average numbers of immunogenic epitopes from Gag, Pol, and Env are 3.44, 2.08, and 1.22, respectively, mirroring the trends of CTL A-list epitope densities (0.16, 0.10, and 0.05). The density calculation simply divides the number of known CD8 T-cell epitopes in each protein by its sequence length, providing a length-normalized measure of how many epitopes each protein typically contributes. Thus, the relative contributions of all three proteins align with known immunogenicity patterns.

The number of immunogenic epitopes and immunogenic epitope pairs for tHIVconsvX, the epitopes from Gaiha et al., and the Murakowski immunogen are reported in S3 Table and S9 Fig. Among these, the epitopes from Gaiha et al. yield the fewest immunogenic epitopes, totaling 6, while tHIVconsvX generates the highest count of 16. These estimates are reasonable, particularly when considering that individuals with chronic HIV infections typically target an average of 20 epitopes [34,45]. The primary factor influencing the number of immunogenic epitopes is the quantity of epitopes present, which also relates to the length of the immunogen. tHIVconsvX and the Murakowski immunogen incorporate longer blocks, encompassing more potential epitopes that can be targeted by a wider range of HLA types. However, this expansion comes at the cost of possibly directing the immune response away from conserved or multidimensionally conserved epitopes. In contrast, our immunogen and the epitopes from Gaiha et al. feature fewer immunogenic epitopes. Our immunogen focuses on multidimensionally conserved epitopes, while Gaiha et al. prioritizes highly networked epitopes. Despite this, our immunogen still contains at least one immunogenic epitope pair for every haplotype in the sampled Caucasian population, and at least one highly networked epitope from Gaiha et al. is immunogenic to every haplotype as well.

We further deconstructed the immunogenicity of the other immunogens by protein (S10–S12 Figs). For tHIVconsvX, the immunogenic coverage is derived exclusively from Pol and Gag, with no immunogenic epitopes contributed by Env. The Murakowski immunogen shows a heavy reliance on Gag, while the epitopes from Gaiha et al. are more uniformly distributed across proteins but with smaller numbers of immunogenic epitopes.

## 3. Discussion

Multiple avenues are being explored in the pursuit of an HIV vaccine. On one front, researchers are directing their efforts towards producing vaccines that elicit broadly neutralizing antibodies to bind conserved, functional sites on Env and prevent infection [8,10,46,47]. On the other front, the focus is on eliciting cytotoxic T-cells to kill infected host cells [48], which could contribute to controlling viral load to low levels. This approach is supported by observations that certain individuals, referred to as elite controllers, possess effective T-cell responses and naturally maintain low viral loads without therapy [49,50].

The most formidable challenge to creating an effective vaccine is the extreme mutability and replicability of HIV, which allows it to generate escape mutations against an array of immune responses. However, HIV cannot make any possible mutation in order to escape immunity, because the mutations it makes must maintain viral fitness. The crux of an effective T cell-based vaccine strategy lies in selectively targeting regions where it is difficult for the virus to select escape mutations because of the fitness cost, potentially trapping the virus between its own fitness requirements and the induced T-cell response. Fitness can encompass various properties including, but not limited to, structural stability of viral proteins. Regarding structural stability, Gaiha et al. applied network analysis to calculate highly networked epitopes, which are likely important for stability [28]. Other approaches seek to glean insights into fitness from sequence data, assuming that sequences observed more frequently correspond to fitter viruses, and this notion of fitness could relate to structural stability but potentially other properties such as conformational changes upon binding [22]. tHIVconsvX is an immunogen that was designed by determining which regions of the HIV proteome are highly conserved at single residues [14], as it is believed that conserved residues correspond to high fitness cost regions.

Our approach adds an additional layer on top of using single residue conservation. We use the fitness landscape of HIV derived from sequence data to identify multidimensionally conserved regions, which are regions that are both conserved and unlikely to contain compensatory interactions with other parts of the sequence and likely to contain regions where mutations are antagonistic. This approach aims to overcome the challenge that HIV can take advantage of epistatic, compensatory pathways to evolve escape mutations while maintaining its fitness [18].

A limitation of the fitness landscape is that it only considers epistasis within a protein and does not model epistasis across proteins. The primary reason we do not model inter-protein epistasis is that it requires sequence data for the entire proteome of the virus, and the statistics obtained from the available whole proteome sequence data would be inadequate for inference. Attempting to fit parameters across the entire genome would also require a computationally infeasible number of coupling parameters. Furthermore, focusing on intra-protein epistasis is an appropriate approximation because it has been found that intra-protein epistasis is more prominent than inter-protein epistasis [51].

As previously detailed in the Introduction, predictions from the fitness landscape have been shown to be predictive of in vitro experimental measurements of viral replication rates, and measured escape times in humans given knowledge of the targets of T cell responses in each person [19,22,23]. Theoretical studies suggest reasons why the prevalence landscape inferred from sequence data is predictive of intrinsic viral fitness. Despite these past studies, additional tests of whether escape mutations in the regions of our proposed immunogen are inhibited need to be tested in pre-clinical models.

In Murakowski et al., the fitness landscape was used to design an immunogen for the adenovirus vector. Given the constraints imposed by the adenovirus vector, the design necessitated the inclusion of certain regions with low fitness costs, making them potential sites for viral escape. In contrast, our immunogen, influenced by the emergence of mRNA vaccines that do not have the same constraints as the adenovirus vector, exhibits greater selectivity in determining which regions are included in the immunogen. As a result, these regions are predicted to be more constrained, making them candidates for an immunogen that elicits T cell responses where escape may be costly. We also considered the immunogenicity of our immunogen in the Caucasian population and show that our immunogen provides broad coverage in the population. Specifically, every haplotype targets at least one epitope pair in our immunogen.

The T-cell response elicited by mRNA vaccines appears promising, as the frequency of SARS-CoV-2 specific CD8^+^ T-cells in response to SARS-CoV-2 mRNA vaccines is similar to the frequencies in response to adenovirus-vectored vaccines [52] and inactivated vaccines [53]. Furthermore, mRNA has been shown to stimulate effective T-cell responses capable of killing tumor cells and reducing the rate of metastasis [42]. The T-cell response may have been enhanced through signal processing domains included in the mRNA, which could also be included in our immunogen. Another intriguing aspect of the study lies in the repeated boosting of the vaccine at short intervals, often occurring within 3–7 days and totaling up to 20 doses, which is quite different from traditional dosing schemes that are usually spaced at least a month apart and contain up to three doses. For a prophylactic vaccine, such frequent dosing would be impractical. However, this is not the case for a therapeutic HIV vaccine, and exploring various unconventional dosing schemes could prove a promising avenue for further investigation.

Another consideration is that our immunogen contains fragments of HIV proteins which may not fold into the same conformations found in native HIV proteins, rendering any antibodies generated non-neutralizing. Therefore, to avoid diverting immune resources toward a non-protective antibody response, we limited the length of our blocks to minimize HLA class II presentation. This approach could shift the immune response toward the class I pathway, thereby emphasizing the T-cell response. In addition, signal processing domains aimed at enhancing the class I pathway could be incorporated into the immunogen [54]. It may also be possible to provide our immunogen alongside an antibody-inducing vaccine to generate effective neutralizing antibody responses. Neutralization by broadly neutralizing antibodies is the most direct approach, but recent work has also shown that the generation of autologous antibodies that target multiple HIV epitopes may contribute to viral control upon administration of broadly neutralizing antibodies [55–57], possibly offering a parallel and complementary pathway to control.

An enduring challenge with HIV is the latent reservoir [58], which periodically reactivates and enables the virus to rebound in the absence of therapy. This is the primary issue requiring people living with HIV to be on antiretroviral therapy for their lifetimes. Even if a T-cell response effectively manages the viral population, it must be sustained to address periodic reactivation. In the absence of any antigenic stimulus, the T-cell population and its associated control over the viral population are expected to wane. However, during chronic infection, this could be mitigated by continuous antigen sourced from the viral reservoir. A potential concern is whether the viral antigens could divert the T-cell response from multidimensionally conserved sites to those that are typically immunodominant in a person with a given HLA haplotype, which would render the strategy less effective.

Evolutionary simulations of viral escape could provide insight into the impact of these effects. Such simulations have been performed in the context of HIV T-cell responses to predict viral escape times given the initial T-cell response [19] and to study escape dynamics under immune pressure from autologous neutralizing antibodies [55]. In the context of a designed T-cell immunogen, such simulations could involve simulating personalized viral evolution in individuals with particular viral sequences and HLA types, using the fitness landscape and immunogenicity predictors to model escape pathways. Furthermore, as predictive models improve and more data becomes available, we believe in silico comparisons of different vaccine candidates using artificial intelligence tools will become increasingly feasible. Such advanced approaches would require incorporating factors such as the magnitude of T-cell responses to specific epitopes, the fitness costs of escape mutations, and the probability and kinetics of viral evolution.

## 4. Methods

### 4.1 Calculation of the pairwise fitness cost

First, consider calculating the fitness cost of a single epitope for simplicity. E(s), calculated from Equation 1, is referred to as the energy of a sequence s because of its analogy to the Boltzmann probability in statistical mechanics, where the exponential of the energy is proportional to the probability. To consider the fitness cost of an epitope, let the sequence s be divided into a portion $s_{e}$, which contains the epitope, and $s_{r}$**,** which is the sequence background or the rest of the sequence.

If we make a single mutation in $s_{e}$, then the epitope becomes $s_{e}{\;}^{\prime }$. The fitness cost of making a single mutation is:


$$\begin{array}{c} \begin{array}{c} \delta E\left({𝐬}_{𝐞}^{\prime },{𝐬}_{𝐞}\right)=\sum_{{𝐬}_{𝐫}}\left(E\left({𝐬}_{𝐫},{𝐬}_{𝐞}^{\prime }\right)-E\left({𝐬}_{𝐫},{𝐬}_{𝐞}\right)\right)\text{exp}\left(-E\left({𝐬}_{𝐫},{𝐬}_{𝐞}\right)\right) \end{array} \end{array}$$ (6)

The fitness cost is essentially the difference in energy between the mutated and unmutated sequence, with an additional averaging over all possible sequence backgrounds for the reasons mentioned in Section 2.1.1. The various sequence backgrounds are generated through a Monte Carlo procedure and are weighted according to the probabilities with which they are in circulation, given by the exponential of the energy. With the fitness cost of a single mutation, we can calculate the fitness cost of an epitope by performing an average over all epitope mutations that weights lower fitness cost pathways more heavily:


$$\begin{array}{c} \begin{array}{c} \Delta E=\frac{\sum_{s_{e}^{{\;}^{\prime }}}\delta E\left(s_{e}^{{\;}^{\prime }},s_{e}\right)w\left(s_{e}^{{\;}^{\prime }}\right)}{\sum_{s_{e}^{{\;}^{\prime }}}w\left(s_{e}^{{\;}^{\prime }}\right)} \end{array} \end{array}$$ (7)

$$\begin{array}{c} \begin{array}{c} w\left(s_{e}^{{\;}^{\prime }}\right)=\text{exp}\left(-\delta E\left(s_{e}^{{\;}^{\prime }},s_{e}\right)\right) \end{array} \end{array}$$ (8)

The calculation of the pairwise fitness cost can be done by considering $s_{e}$ to be a pair of epitopes instead of a single epitope, and $s_{e}{\;}^{\prime }$ to be a pair of mutations in $s_{e}$ instead of a single mutation.

## Supporting information

S1 Text Calculation of consensus fraction from LANL HIV sequences. (DOCX)

S2 Text Contributions to ΔE_12_, ΔE_1_, and ΔE_2._ (DOCX)

S1 Table HIV proteins for which the fitness landscape was inferred. (DOCX)

S2 Table Nucleotide and amino acid sequences of the joined immunogen using regions of the HXB2 HIV strain corresponding to our identified blocks. (DOCX)

S3 Table The average number of immunogenic epitopes and immunogenic epitope pairs in our immunogen and previous immunogens. (DOCX)

S1 Fig ROC curve of the immunogenicity predictor evaluated on HIV epitopes from the IEDB database. (TIF)

S2 Fig HXB2 sequence for Pol highlighting the location of multiple immunogens. (TIF)

S3 Fig HXB2 sequence for Env highlighting the location of multiple immunogens. (TIF)

S4 Fig HXB2 sequence for Gag highlighting the location of multiple immunogens. (TIF)

S5 Fig HXB2 sequence for accessory and regulatory proteins highlighting the location of multiple immunogens. (TIF)

S6 Fig Distributions of fields and couplings for entire proteins and blocks in our immunogen. (TIF)

S7 Fig Comparison of inter-epitope interactions between entire proteins and blocks in our immunogen. (TIF)

S8 Fig Distribution of immunogenic epitopes and pairs per protein for blocks in our immunogen. (TIF)

S9 Fig Distribution of immunogenic epitopes and pairs for other immunogens. (TIF)

S10 Fig Distribution of immunogenic epitopes and pairs per protein for tHIVconsvX. (TIF)

S11 Fig Distribution of immunogenic epitopes and pairs per protein for Gaiha et al. epitopes. (TIF)

S12 Fig Distribution of immunogenic epitopes and pairs per protein in the Murakowski immunogen. (TIF)
