# Supplementary material for: Design of an immunogen containing multidimensionally conserved and immunogenic parts of the HIV proteome
Source: PLoS Comput Biol. 2026 Aug 14;22(8):e1014632. doi: 10.1371/journal.pcbi.1014632 (PMC13492788; doi:10.1371/journal.pcbi.1014632)
Supplement: Supplementary file 1 [file pcbi.1014632.s001.docx]

**Calculation of consensus fraction from LANL HIV sequences**

Pre-aligned HIV-1 subtype B sequences for Env, Gag, Nef, Pol, Rev, Tat, Vif, Vpr, and Vpu were obtained from the LANL database ([*https://www.hiv.lanl.gov/*](https://www.hiv.lanl.gov/)) using the sequence search interface. 59537 sequences were obtained for Env, 25710 for Gag, 30994 for Nef, 17732 for Pol, 22060 for Rev, 20223 for Tat, 24300 for Vif, 23914 for Vpr, and 26572 for Vpu. The HXB2 reference sequence was included with each alignment, and residues with gaps in the HXB2 sequence were removed from each protein alignment. Then, the consensus amino acid at each residue was determined, and the fraction of amino acids that matched the consensus was calculated and used to determine the conservation of each residue on the HXB2 sequence.
