# Supplementary material for: Design of an immunogen containing multidimensionally conserved and immunogenic parts of the HIV proteome
Source: PLoS Comput Biol. 2026 Aug 14;22(8):e1014632. doi: 10.1371/journal.pcbi.1014632 (PMC13492788; doi:10.1371/journal.pcbi.1014632)
Supplement: Supplementary file 2 [file pcbi.1014632.s002.docx]

**Contributions to** $\boldsymbol{\Delta}\boldsymbol{E}_{\boldsymbol{12}}$**,** $\boldsymbol{\Delta}\boldsymbol{E}_{\boldsymbol{1}}$**, and** $\boldsymbol{\Delta}\boldsymbol{E}_{\boldsymbol{2}}$

The energy of a sequence with a mutated epitope is:

$$E_{1}^{'}=-\sum h_{r}-\sum h_{e_{1}^{'}}-\sum J_{rr}-\sum J_{re_{1}^{'}}-\sum J_{e_{1}^{'}e_{1}^{'}}$$

where $e_{1}^{'}$ indicates the mutated epitope and $r$ indicates the rest of the sequence. The energy contains contributions from the fields of the residues as well as couplings between pairs of residues.

To clearly illustrate the algebraic contribution of inter-epitope couplings, the derivation below approximates the fitness cost as the energy difference relative to a fixed background. Note that the actual fitness cost values reported in the Results section are calculated by rigorously averaging over the ensemble of sequence backgrounds as described in the Methods.

If the unmutated epitope is indicated by $e_{1}$, then the change in energy or fitness cost is given by the expression below.

$$\Delta E_{1}=-\sum\left( h_{e_{1}^{'}}-h_{e_{1}} \right)-\sum\left( J_{re_{1}^{'}}-J_{re_{1}} \right)-\sum\left( J_{e_{1}^{'}e_{1}^{'}}-J_{e_{1}e_{1}} \right)=-\sum h_{e_{1}^{'}}-\sum J_{re_{1}^{'}}-\sum J_{e_{1}^{'}e_{1}^{'}}$$

where simplifications are made that the fields and couplings of the unmutated consensus sequence are 0. The fitness cost of a single epitope contains couplings within the mutated epitope and couplings between the epitope and the rest of the sequence. Given another epitope $e_{2}$, the sum of the individual fitness costs is

$$\Delta E_{1}+\Delta E_{2}=-\sum\left( h_{e_{1}^{'}}+h_{e_{2}^{'}} \right)-\sum\left( J_{re_{1}^{'}}+J_{re_{2}^{'}} \right)-\sum\left( J_{e_{1}^{'}e_{1}^{'}}+J_{e_{2}^{'}e_{2}^{'}} \right)$$

For a pair of epitopes $e_{1}$ and $e_{2}$, the pairwise fitness cost is

$$\Delta E_{12}=-\sum\left( h_{e_{1}^{'}}+h_{e_{2}^{'}} \right)-\sum\left( J_{re_{1}^{'}}+J_{re_{2}^{'}} \right)-\sum\left( J_{e_{1}^{'}e_{1}^{'}}+J_{e_{2}^{'}e_{2}^{'}} \right)-\sum J_{e_{1}^{'}e_{2}^{'}}$$

The pairwise fitness cost contains couplings within the mutated epitopes, couplings between the epitopes and the rest of the sequence, and couplings between the epitopes. Lastly, note that $\Delta E_{12}-\Delta E_{1}-\Delta E_{2}=-\sum J_{e_{1}^{'}e_{2}^{'}}$, indicating that $\Delta E_{12}-\Delta E_{1}-\Delta E_{2}$ captures the effects of couplings between the epitopes. Negative values of the couplings indicate antagonistic interactions and positive values indicate compensatory interactions, so here negative values of $\Delta E_{12}-\Delta E_{1}-\Delta E_{2}$ indicate compensatory interactions and positive values indicate antagonistic interactions.
