# Supplementary material for: Design of an immunogen containing multidimensionally conserved and immunogenic parts of the HIV proteome
Source: PLoS Comput Biol. 2026 Aug 14;22(8):e1014632. doi: 10.1371/journal.pcbi.1014632 (PMC13492788; doi:10.1371/journal.pcbi.1014632)
Supplement: Supplementary file 3 [file pcbi.1014632.s003.docx]

**Table S1**. Nucleotide and amino acid sequences of the joined immunogen using regions of the HXB2 HIV strain corresponding to our identified blocks. The HXB2 strain differs from the consensus sequence in two residues specified in the main text.

| Gene | Protein |
| --- | --- |
| pol | prot, p51 RT, p31 int |
| gag | p17, p24, p7, p6 |
| env | gp120, gp41 |
| nef | nef |
| vif | vif |
| vpr | vpr |
| tat | tat |
| vpu | vpu |
| rev | rev |
