# Supplementary material for: Design of an immunogen containing multidimensionally conserved and immunogenic parts of the HIV proteome
Source: PLoS Comput Biol. 2026 Aug 14;22(8):e1014632. doi: 10.1371/journal.pcbi.1014632 (PMC13492788; doi:10.1371/journal.pcbi.1014632)
Supplement: Supplementary file 5 [file pcbi.1014632.s005.docx]

**Table S3**. The average number of immunogenic epitopes and immunogenic epitope pairs in our immunogen and previous immunogens. The average is calculated across the Caucasian population using haplotypes and haplotype frequencies from the Allele Frequency Net Database.

| Immunogen | Average number of immunogenic epitopes | Average number of immunogenic epitope pairs |
| --- | --- | --- |
| This work | 7 | 8 |
| Murakowski et al. (*1*) | 13 | 23 |
| Gaiha et al. epitopes (*2*) | 6 | 5 |
| tHIVconsvX (*3*) | 16 | 46 |
